# Clinical Improvement and P63-Deficiency Correction in OLP Patients After Photobiomodulation

**DOI:** 10.3390/dj12110338

**Published:** 2024-10-22

**Authors:** Maria Zaharieva Mutafchieva, Milena Nenkova Draganova, Blagovesta Konstantinova Yaneva, Plamen Ivanov Zagorchev, Georgi Tomchev Tomov

**Affiliations:** 1 Department of Periodontology and Oral Mucosa Diseases, Faculty of Dental Medicine, Medical University of Plovdiv, 4000 Plovdiv, Bulgaria; blagovesta.yaneva@mu-plovdiv.bg; 2 Department of Medical Biology, Faculty of Medicine, Medical University of Plovdiv, 4000 Plovdiv, Bulgaria; 3 Research Institute, Medical University of Plovdiv, 4000 Plovdiv, Bulgaria; plamen.zagorchev@mu-plovdiv.bg; 4 Department of Medical Physics and Biophysics, Faculty of Pharmacy, Medical University of Plovdiv, 4000 Plovdiv, Bulgaria; 5 Department of Healthcare and Social Work, New Bulgarian University, 1618 Sofia, Bulgaria; dr.g.tomov@gmail.com

**Keywords:** oral lichen planus, p63 expression, molecular biomarkers, photobiomodulation, PBM, 810 nm diode laser, oral mucosal lesions, VAS, TSSS, Treatment efficacy index

## Abstract

**Background:** Oral lichen planus (OLP) is a chronic inflammatory disease associated with the formation of symptomatic lesions in the mouth. P63 is essential for epidermal development and regeneration. Weak expression of this protein has been shown in OLP lesions. Photobiomodulation (PBM) therapy has been reported to reduce OLP symptoms, but its ability to correct the molecular perturbations of the disease has not been studied. This study aimed to evaluate the efficacy of PBM in OLP treatment by evaluating changes in p63 expression and their association with clinical response. **Methods:** Twenty OLP patients underwent PBM with a diode laser (810 nm), (0.50 W, 30 s, 1.2 J/cm^2^), 3 times weekly for a month. The treatment efficacy index (EI) was calculated based on pain-level values and clinical scores of lesions before and after therapy. Biopsies were taken before and after therapy, analyzed immunohistochemically for p63 expression, and compared with 10 healthy controls. **Results:** P63 levels in OLP lesions were significantly lower than those in normal oral mucosa. After treatment, the pain level and clinical scores of the lesions decreased significantly. The calculated EI showed PBM effectiveness in 90% of cases. Increased p63 positivity and staining intensity were observed after therapy. **Conclusions:** The established p63 deficiency in OLP lesions is likely an important molecular mechanism in the pathogenesis of the disease. Laser irradiation at 810 nm increased p63 expression to a level close to that found in the healthy epithelium and significantly improved the symptoms and clinical signs of OLP. All of this determines the effectiveness of PBM therapy in the management of OLP.

## 1. Introduction

### 1.1. Clinical Characteristics of OLP

Oral lichen planus (OLP) is a chronic inflammatory disease leading to the formation of symptomatic lesions in the oral cavity and worsening the quality of life of patients. The average prevalence of this condition worldwide ranges from 2 to 5%, affecting predominantly women in their fifth and sixth decades of life [1]. Its hallmark features include multiple lesions with bilateral distribution, pain, and chronic, recalcitrant course [2]. Pathognomonic findings in the oral cavity are Wickham striae, representing white keratotic lines in a lace-like pattern. The latter are the presenting feature of the most common, classic form of OLP—the reticular form. Hyperkeratotic changes can sometimes appear as small papules of 0.5–1 mm (papular form) or more homogeneous plaques that resemble those of leukoplakia (plaque-like form). The atrophic form is characterized by diffuse, erythematous areas, surrounded by a hyperkeratotic periphery. It often affects the gingiva in the form of desquamative gingivitis. In more severe cases, erosions of various sizes, covered by a fibrin coating (erosive form) or even blisters (bullous form) can be seen in the oral cavity. Important for the diagnosis is the detection of keratotic striae on the periphery of all these lesions. The six clinical forms of OLP can be grouped into keratotic (reticular, papular, plaque-like) and non-keratotic forms (atrophic, bullous, and erosive forms). The latter are associated with more intense pain.

As there is a group of disorders, known as oral lichenoid lesions (OLLs), that resemble OLP [3], the clinical diagnosis of the disease should always be confirmed by pathomorphological examination. Histological criteria for OLP were first proposed by the World Health Organization (WHO) in 1978 [4], then modified by Van der Meij in 2003 [5] and last updated in 2016 by the American Academy of Oral and Maxillofacial Pathology to include band-like, predominately lymphocytic infiltrate, confined to the epithelium–*lamina propria* interface; basal cell liquefactive (hydropic) degeneration; lymphocytic exocytosis; the absence of epithelial dysplasia; and the absence of verrucous epithelial architectural change [3]. In diagnostically difficult cases, immunofluorescence is indicated to reveal shaggy fibrinogen deposition at BMZ and colloid bodies, which is typical of OLP [3].

### 1.2. Etiopathogenesis

Lichen planus is an immune-mediated condition of unknown etiology in which CD8+ T-Ly destroy basal keratinocytes of the stratified epithelium through activation of a cell death program (apoptosis). On the other hand, histological findings such as epithelial hyperplasia with hyperkeratosis, parakeratosis, and acanthosis, which are typical of the disease, indicate pathologically altered proliferation and differentiation and thus raise the question of a presumably disturbed balance between cell proliferation and cell death. Moreover, after the first announce of the malignant potential of OLP lesions in 2005 [6], according to the current updated consensus report from an international seminar on nomenclature and classification, convened by the WHO Collaborating Centre for Oral Cancer, 2021, OLP is still included in the group of oral potentially malignant disorders (OPMDs) [7]. The rate of malignant transformation (MT) of OLP is about 1% [8]. The erosive form of OLP is more likely to develop into oral squamous cell carcinoma (OSCC) than the others [8]. Location (tongue > buccal > buccal mucosa) and extent of involvement also influence MT risk [8]. Considering all this information, it is understandable why the scientific interest nowadays is focused on the investigation of biomarkers profoundly associated with cell cycle control, apoptosis, proliferation, cell differentiation, and cancer development in OLP lesions. P63 is such a marker [9,10].

### 1.3. P63 Protein

P63 is a transcription factor of the p53 family, along with p53 and p73. All members of the family share structural similarities (as they all have three main domains: the N-terminal Trans-activation domain (TAD), the DNA-binding domain (DBD), and the oligomerization domain (OD), and therefore have lots of overlapping functions [11].

P63 is crucial for the development of tissues of ectodermal origin—skin, oral mucosa, hair follicles, teeth, salivary, lacrimal, and mammary glands. This statement is supported by the results from experimental studies with mice. P63-null mice die at birth, displaying an absence of normal stratified epithelium, including epidermis and epidermal appendages, truncated limbs, and craniofacial malformations [12,13]. Additionally, germline mutations of p63 in humans cause six rare autosomal-dominant developmental syndromes associated with ectodermal dysplasia, orofacial clefts, and abnormalities in limb development [14,15]. In a post-developmental context, p63 is required for both proliferation and differentiation of developmentally mature keratinocytes [16]. Experimental knockdown of p63 expression resulted in severe tissue hypoplasia and both stratification and differentiation defects [16]. P63 maintains epithelial integrity by regulating the expression of different markers, providing stable adhesion of basal keratinocytes to the underlying basement membrane and thus preventing anoikis—a form of cell death [14]. The p63 protein is also involved in cell cycle arrest, apoptosis, and cell senescence [10,11]. The fundamental role of p63 in epithelial development and renewal may explain the altered levels of this protein in tumors of epithelial origin. Significant overexpression of p63 has been reported in 88% of squamous carcinomas [14].

There are two main classes of p63 isoforms, generated by alternative promoters. Transcripts generated by promoter 1 (P1) encode the full-length (long) isoform—TAp63, which contains all three domains (TAD, DBD, and OD). An alternate promoter (P2) produces transcripts encoding the short ΔNp63 isoform that lack the N-terminal transactivation domain [14,17]. Alternative splicing generates multiple variants that differ in their C-termini (α, β, γ, δ, and ε) for both TAp63 and ΔNp63 [14]. The different isoforms of p63 have diverse, often opposing functions. TAp63 variants are prevalent in the dermal stem cells, heart, testis, kidney, thymus, brain, and cerebellum, while ΔNp63 transcripts are mainly restricted to the epithelium, developing in the teeth, kidney, spleen, and thymus [11,14]. ΔNp63 is essential for epidermis development, promotes cell proliferation and self-renewal, inhibits apoptosis and senescence, and is an oncogenic driver in squamous cell carcinoma [11,17]. In contrast to ΔNp63, TAp63 isoforms can induce cell quiescence, cellular senescence, and apoptosis, and inhibit tumor formation and metastasis, suggesting that TAp63 is a tumor suppressor [11,14]. ΔNp63 isoform was found to confer negative effects on TAp63 transcriptional activity [11].

### 1.4. P63 Expression in Patients with OLP

The role of p63 in the pathogenesis of OLP has been actively speculated on since Ebrahimi M et al. detected circulating antibodies against this protein in the serum of five patients with OLP [18,19]. Given the autoimmune genesis of the disease, it could be suggested that the trigger of the immune aggression is epidermal factor p63. What is more, one of the isoforms, ΔNp63a, is the antigen eliciting autoimmunity in patients with chronic ulcerative stomatitis (CUS) [18,20]. CUS is a disorder that resembles oral lichen planus both clinically and histologically and that, according to some authors [18,20], should be regarded as a variant of lichen planus rather than as a distinct entity. The fact that ΔNp63a is the target of autoantibodies in a condition very similar to OLP explains the scientific interest in investigating the role of p63 in the latter. The conducted studies almost unanimously demonstrated pathologically decreased expression of p63 in the epithelium of OLP lesions [21,22,23], indicating the significance of p63 deficiency in the observed tissue disturbances. However, to the best of our knowledge, there is currently no clinical trial evaluating the effects of some of the OLP treatment modalities on p63 expression.

### 1.5. Treatment Modalities of OLP

The management of patients with OLP is often challenging for the following reasons:

1. The symptoms of oral pain and/or burning interfere with food and beverage intake, make speech and social communication difficult, increase patients’ anxiety, impair their psycho-emotional health, and generally worsen the quality of life (QoL) of the patients. 2. The lesions tend to persist for months or even years. 3. Since the disease’s trigger is unidentified, the administered therapy is not curative. 4. Application of the topical treatment forms is hindered by saliva. 5. There are frequent relapses. 6. All pharmacological treatment forms used in OLP are associated with adverse effects [2]. Priority shall be given to topical agents. Corticosteroids (clobetasol propionate 0.05%, triamcinolone, betamethasone, fluocinolone) are the first line of choice. Calcineurin inhibitors (tacrolimus and pimecrolimus), retinoids (tretinoin 0.05%), and other immunosuppressants (cyclosporine) are also used with varying degrees of utility [2]. Systemic therapy is indicated in cases that are recalcitrant and do not respond satisfactorily to topical agents. Systemic corticosteroids (prednisolone or methylprednisolone) are the most effective treatment modality for patients with multisite recalcitrant lesions. Oral cyclosporine, oral retinoids (acitretin), and methotrexate are less commonly used. A number of new pharmacological and non-pharmacological treatment methods have been proposed, including laser therapy (see below), platelet-rich plasma, topical thalidomide, topical hyaluronic acid, piperine extracted from black pepper, aloe vera gel, topical and oral curcuminoids, zinc (oral and topical), selenium, propolis, topical tocopherol, and probiotics. Randomized controlled trials are needed to establish the efficacy and safety of these modalities [8].

### 1.6. Laser Therapy in OLP

The utilization of different kinds of dental lasers in OLP management has demonstrated promising treatment results. There are two approaches of laser therapy—ablative and non-ablative. Ablative laser treatment is associated with either an excision of the affected tissue or its ablation. The latter is a particularly beneficial method for OLP patients, since the lesions are usually multiple and disseminated, and deep excisions may lead to the formation of multiple scars. Efficient elimination of the oral lesions and symptomatic relief were reported after ablation using a CO_2_ laser [24], an Er:YAG laser [25], and a diode laser (810 nm) [26]. The non-ablative approaches are photobiomodulation (PBM) [27] and anti-microbial photodynamic therapy (a-PDT) [28,29]. PBM is a modulation of the cell metabolism and molecular interaction utilizing a low-intensity laser or light-emitting diode (LED) light, resulting in several beneficial effects, such as analgesic [30], anti-inflammatory [27], and healing [31,32,33] effects. Different laser types (diode laser, Nd–YAG, He–Ne laser, etc.) with a wavelength window of between 630 nm and 1064 nm and wide heterogeneity in the laser parameters have been used in studies to achieve the effects of biostimulation. Given its ability to minimize pain, eliminate inflammation, and promote tissue regeneration, PBM therapy has emerged as a promising treatment option for OLP patients. Over the past three decades, a great number of articles have been published addressing the utility of this therapy in OLP [34,35,36]. The study conducted by Del Vecchio et al. analyzing 44 studies related to the topic (five systematic reviews, three narrative reviews, three case reports, one ex vivo study, four in vitro studies, four in vivo studies, four clinical trials, twelve case series, and eight randomized clinical trials) determined that PBM laser treatment allowed for excellent management of OLP lesions [36]. Later, in 2022, another systematic review article evaluated the effectiveness of PBM in the atrophic–erosive form of OLP by extracting and summarizing data from seven related studies, and concluded that this type of therapy successfully improves the signs and symptoms of these lesions with no known side effects [34]. Furthermore, multiple studies have compared the effectiveness of corticosteroid therapy (CS) and PBM in the management of OLP and demonstrated similar results, with no difference between the two groups, with the latter being favored due to fewer or no adverse effects being known [37,38,39]. The general consensus is that PBM is an effective therapeutic alternative to conventional OLP treatments and can be used in cases where corticosteroids are ineffective or contraindicated due to comorbidity. However, the latter statement is primarily based on clinical results. Almost all of the studies cited above used a visual analog scale (VAS) and clinical sign scoring systems (mainly Thongprasom) [34] to demonstrate pain relief and improvement in disease signs after PBM, but the precise molecular mechanisms remain elusive. In this regard, Wang T et al. (2024) demonstrated the ability of PBM to upregulate the expression of p63, which was accompanied by enhanced reepithelialization and accelerated wound healing in rats [40]. Elevated p63 levels resulting in significantly increased proliferation of keratinocytes after low-intensity laser irradiation were reported also by Sperandio FF et al. (2015) [33]. Hence, the aim of this study was to assess PBM efficacy in OLP management by evaluating p63 expression changes and relating them to clinical response.

## 2. Materials and Methods

### 2.1. Study Design

This was a prospective clinical observational study. Patients with signs and symptoms consistent with oral lichen planus were selected among those attending the Department of Periodontology and Oral Mucosal Diseases of the Faculty of Dental Medicine, Medical University of Plovdiv, Bulgaria. A biopsy was then taken for histological confirmation of the diagnosis. Thus, a group consisting of 20 patients of both genders aged ≥18 years diagnosed with different forms of symptomatic OLP based on clinical examination and histological analysis was formed. Glass slides from the stored paraffin blocks were prepared for immunohistochemical analysis of the expression of p63. To determine the effects of p63 expression levels on the proliferation process, we looked for an association with the proliferative marker Ki-67 [41], expressed in the same 20 OLP patients.

Additionally, to compare the levels of p63 in OLP with those in healthy mucosa, biopsies were taken from 10 volunteers who presented with no lesions in the oral cavity and no anamnesis for previous oral mucosa diseases. Then, all OLP patients underwent PBM with an 810 nm diode laser for a month, with the treatment protocol described below. After completion of the therapy, control biopsies were collected to assess the obtained p63 expression changes.

The clinical examination by oral pathology specialist, biopsy procedures, treatment interventions, and follow-up visits of the patients were conducted at the Department of Periodontology and Oral Mucosa Diseases, Faculty of Dental Medicine, Medical University of Plovdiv. Immunohistochemical examination was performed at the Department of Medical Biology, Faculty of Medicine, Medical University of Plovdiv.

As our study is an observational study and not a randomized controlled trial (RCT), we allocated two independent, experienced assessors for variable evaluation and data collection to minimize the interobserver variability and bias.

The study was conducted in accordance with the Declaration of Helsinki. The research protocol was approved by the Ethics Committee of the Medical University of Plovdiv (R3716/07.10.2014).

#### 2.1.1. Research-Focused Questions

- Is the expression of the epidermal factor p63 pathologically altered in the epithelium of OLP lesions?

And if “YES”

- Can immunohistochemistry analysis of p63 levels add value in understanding the pathogenesis of the disease?
- Is PBM therapy able to correct the established molecular disturbances?

#### 2.1.2. Research Contingent

- Twenty patients, clinically and histologically diagnosed with different forms of oral lichen planus. All patients were first diagnosed with OLP, with no history of previous biopsy or treatment.
- Ten healthy volunteers.

Informed written consent was obtained from all participants in the study.

#### 2.1.3. Inclusion Criteria

- Patients with any kind of clinical forms of OLP—reticular, papular, plaque-like, atrophic, bullous, or erosive forms.
- OLP patients of both genders (female and male) aged ≥18 years old.
- Patients who reported pain or some degree of oral discomfort.
- Patients with a histologically confirmed diagnosis of OLP—the WHO’s modification of the criteria of Van der Meij [5] were applied to obtain a histological diagnosis of OLP: well-defined, band-like, predominately lymphocytic infiltrate, confined to the epithelium–lamina propria interface; basal cell liquefactive (hydropic) degeneration; and the absence of epithelial dysplasia.
- Patients agreed to comply with the study design.
- Age- and sex-matched healthy volunteers (without OLP or any other mucosal lesions) who needed any surgical procedure associated with tissue excision (mainly tooth extraction). Only tissues with no visible signs of inflammation were collected. Histological examination was then performed to confirm normal oral mucosa (NOM) (control group).

#### 2.1.4. Exclusion Criteria

- Patients who have received local or systemic therapy (corticosteroids, non-steroidal anti-inflammatory drugs, or immunosuppressive agents) for this or any other autoimmune, inflammatory, or allergic comorbidity in the last month due to the risk of compromising the results.
- Patients with clinical presentation that resembles a lichenoid reaction—unilateral lesions with a direct topographic relationship to amalgam fillings/dental restoration(s), a history of a temporal association between the introduction of a drug and the onset of the disease, or history of past transplantation.
- Patients with dysplastic features

### 2.2. Incisional Biopsy with ER:YAG Laser

A biopsy was taken from all 20 OLP patients and 10 healthy controls using an Er:YAG laser with the following parameters: pulse mode; 35 Hz; 7 W; 200 mJ. Tissue samples were taken along the borders of the lesions, avoiding areas with excess fibrin coating as well as ulcerative fields due to epithelial absence. Biopsies were stored in 10% formalin at neutral pH (6.8–7.2) (biopsy:solution ratio 1:10) until being embedded in paraffin. Glass slides were then prepared for histological analysis (hematoxylin and eosin (H&E) staining) and for immunohistochemistry.

### 2.3. Treatment Protocol

The work group (OLP patients) was subjected to PBM therapy. A “Syneron” diode laser (medical/laser class IV; beam profile—Gaussian) with a wavelength of 810 nm was used. The lesions were irradiated with an intra-oral device from a distance of 2 mm, with a beam spot size of 12.5 mm and with overlapping irradiated points to cover the entire surface. The treatment sessions were repeated three times per week with an interval of a day for 4 weeks, with a total of 12 treatment sessions. PBM dosimetry: CW; 0.5 W; 30; 1.2 J/cm^2^; 15 J; 0.04 (W/cm^2^). Anatomical location—buccal mucosa, tongue, labial mucosa, gingiva, and hard palate.

### 2.4. Clinical Result Assessment Tools

- Treatment efficacy index (EI) [24].

Treatment efficacy assessment criteria are the changes obtained in a patient’s symptoms scores and in the clinical sign scores of the lesions after PBM.

The total score (TS), as a sum of the VAS score and the TSSS score, was calculated for each patient before (TS0 = VAS0 + TSSS0) and after (TS1 = VAS1 + TSSS1) therapy. The treatment efficacy index (EI) was then calculated as a percentage (%) by the following formula:

EI = TS0 − TS1/TS0 × 100

The EI was categorized into five rank scale as follows:

⮚ Healed: EI = 100%;
⮚ Marked improvement: 75% ≤ EI < 100%;
⮚ Moderate improvement: 25% ≤ EI < 75%;
⮚ Mild improvement: 0 < EI < 25%;
⮚ No improvement: EI = 0.

Data about patient pain intensity (measured by the Visual Analogue Pain Rating Scale (VAS)) and clinical sign scores of the lesions (assessed by the Thongprasom Sign Scoring System (TSSS)) needed to be recorded prior to calculation of the treatment efficacy index (EI), where:

⮚ VAS0 and VAS1 signify the pain score before and pain score after therapy, respectively;
⮚ TSSS0 and TSSS1 signify clinical sign scores for lesions before and clinical sign scores for lesions after therapy, respectively;
⮚ VAS [42] was used to measure the level of pain from 0 (“no pain”) to 10 (“extremely severe pain”), and the values were then categorized as follows: score 0: (VAS = 0); score 1: (0 < VAS ≤ 3); score 2: (3 < VAS ≤ 7); score 3: (7 < VAS ≤ 10).
⮚ Clinical sign scores of the lesions according to the TSSS are as listed: score 0: no lesions; score 1: white striae only; score 2: white striae with atrophic area < 1 cm^2^; score 3: white striae with atrophic area > 1 cm^2^; score 4: white striae with erosive area < 1 cm^2^; score 5: white striae with erosive area > 1 cm^2^ [24,43].

### 2.5. Immunohistochemical Examination

Altogether, 50 glass slides (20 from OLP patients’ biopsies at baseline, 20 from the OLP patients’ biopsies after PBM therapy, and 10 from healthy subjects’ biopsies) were prepared for immunohistochemical analysis of p63 expression. The examination was conducted using the biotin–streptavidin peroxidase method.

Immunohistochemical staining was performed on 4 μm-thick paraffin sections fixed on poly-L-lysine-coated slides. After deparaffinization in xylene and rehydration in ethanol, the slides were immersed in 0.01 M citrate buffer (pH 6.0) for 20 min in a 95 °C water bath for antigen retrieval. Then, endogenous peroxide was blocked by 3% H_2_O_2_, followed by a protein block (Bio SB-mouse/rabbit polydetector HRP/DAB kit (Cat.N: BSB 0201S)). The sections were then incubated with monoclonal antibodies against p63 (monoclonal Ra Hu p63 protein, clone: EP174, Bio SB) for two hours. Then, the samples were incubated with biotinylated secondary antibody for one hour and with streptavidin-HRP for 30 min at room temperature (RT). Detection of the antigen–antibody reaction was carried out by 3, 3′-diaminobenzidine (DAB). Cell nuclei were counterstained with hematoxylin.

The expression was assessed by the presence of red-brown granular staining. The reaction intensity was measured using a semiquantitative scale: (−) negative result—<5% stained cells; (+) weak expression—5–25% positive cells; (++) moderate expression—25–50% positive cells; (+++) strong expression—staining in > 50% of the cells. A Nicon eclipse Ni-U light microscope was used. P63 demonstrated clear brownish nuclear staining. However, staining intensity may raise questions between positivity and negativity. For cases that could raise doubts, a 2-observer calibration was performed.

Sections from the prostate served as positive controls for the expression of p63. A slide incubated with PBS (phosphate-buffered saline) alone instead of the primary antibody was used as a negative control.

### 2.6. Statistical Analysis

Statistical analysis was performed using SPSS 11.5 Inc. (Chicago, IL, USA) and Excel 7.0 VB for applications in GraphPad Prism 3.0 (GraphPad Software, San Diego, CA, USA). After checking the distribution for normality of the variables, the Wilcoxon matched-pairs test was chosen to determine the difference in pain level and the Mann–Whitney test was chosen to determine the difference in lesion sign scores and p63 levels before and after therapy. The chi-squared test was used to determine the statistically significant difference in p63 expression between patients with OLP and healthy controls, as well as according to clinical form. Correlation between the levels of p63 and the proliferative marker Ki-67 [41] was established by applying the Spearman correlation test. A *p*-value of less than 0.05 was considered statistically significant. The results were presented as mean ± SDM, where the mean is the average value and SDM is the standard deviation of the mean.

## 3. Results

### 3.1. Epidemiology Characteristics

Consistent with the literature, the results from our study demonstrated a female predominance among the OLP contingent, as 85% of the patients were women and only 15% were men. The mean age of the participants was 52.9 years, with two age peaks recorded between 41 and 50 and over 61. Since most of the patients presented with multiple lesions of different types, when determining belonging to a given group of OLP clinical forms, priority was given to the most severe of the those detected. Patient characteristics are summarized in Table 1.

All patients completed the one-month course of PBM therapy. No complications or side effects necessitating adjustments to the treatment protocol or laser parameters were registered.

### 3.2. Treatment Efficacy Evaluation

The effectiveness of the laser therapy was determined by a reduction in the subjective complaints and an improvement in clinical manifestation for each of the patients. The applied Wilcoxon matched-pairs test found a significant reduction in the level of self-reported pain after PBM (*p* < 0.0001). The size and clinical signs of the lesions were also ameliorated significantly from those before treatment (*p* < 0.0001). A total of 95 OLP lesions were registered at baseline. The erosive–atrophic form improved more after laser irradiation compared to the keratotic form. A total of 87% (*n* = 26 out of 30) of the erosions demonstrated a reduction in size to complete resolution. In addition, improvement was achieved in 55% (*n* = 15 out of 27) of atrophic lesions, while only 7.9% (*n* = 3 out of 38) of keratotic striae/plaques disappeared at the end of the treatment course. These data were used to calculate an efficacy index (EI) for PBM therapy.

**Figure 1:**
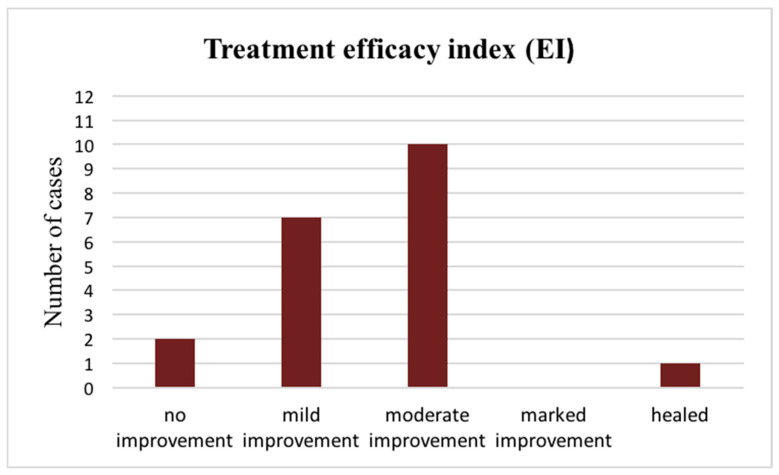
Efficacy index of PBM therapy in patients with OLP.

A total of 90% of the study participants experienced some degree of improvement after PBM therapy. Most of the patients (50%) achieved a moderate recovery. An absence of complaints and complete deletion of the clinical signs of the disease were detected in one patient (Figure 1).

**Figure 2:**
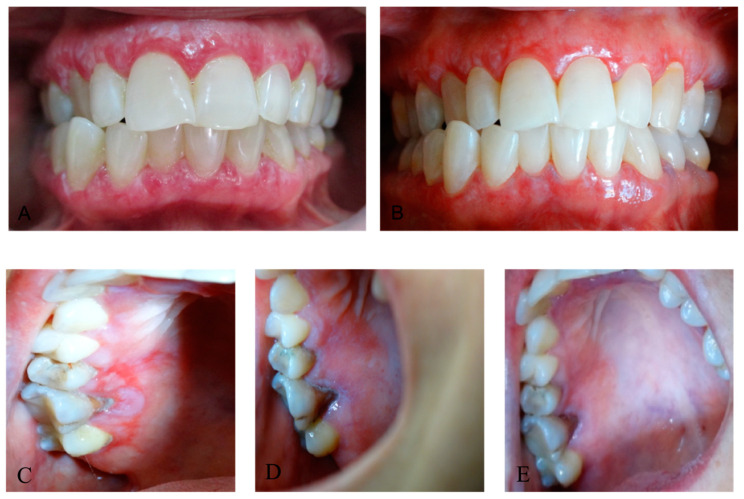
(**A**) Reticular form of OLP on the gingiva with scattered areas of atrophy; (**B**) mild improvement after PBM therapy (**C**) erosive lesion on the hard palate, surrounded by atrophic and keratotic fields; (**D**) healing of the wound at the end of the third week of PBM therapy; (**E**) partial deletion of the keratotic striae at the end of the fourth week of PBM therapy.

Keratotic changes showed less improvement compared with erosive and atrophic lesions (Figure 2).

### 3.3. Immunohistochemical Analysis Results

**Figure 3:**
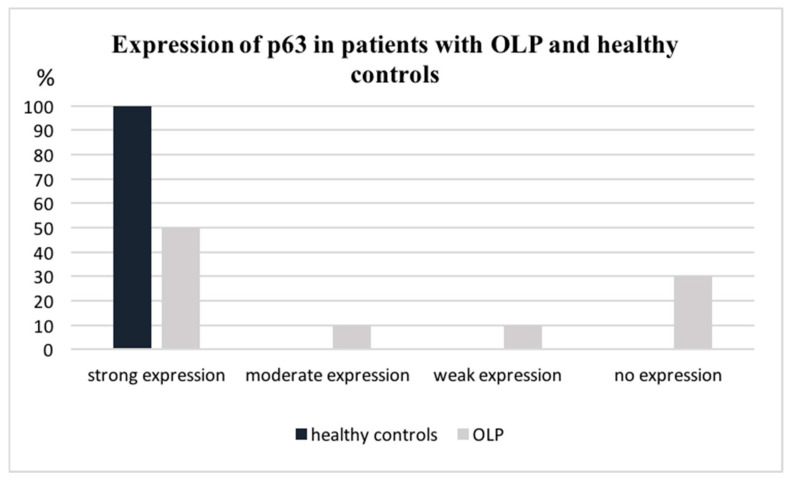
P63 expression in OLP patients (*n* = 20) and healthy controls (*n* = 10).

**Figure 4:**
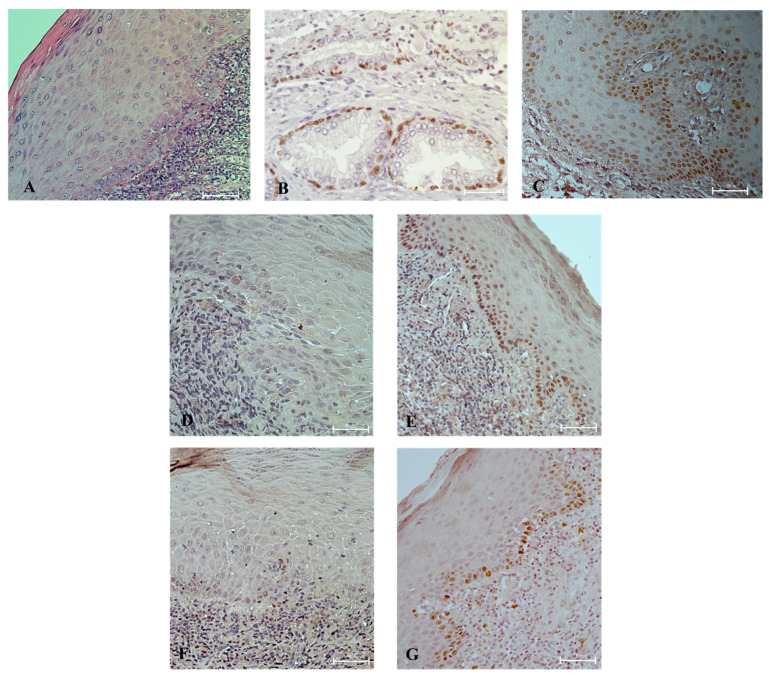
(**A**) Negative control of p63 staining with a slide incubated with PBS instead of primary antibody; (**B**) section from the prostate used as a positive control for the expression of p63; (**C**) strong expression (+++) of p63 in healthy oral mucosa; (**D**) lack of p63 positivity in the epithelium of OLP lesion before therapy (−); (**E**) increase in p63 immunoreactivity after PBM therapy with staining intensity comparable to that in healthy controls (+++); (**F**) corresponding Ki-67 expression for the same patient before (+) and (**G**) after (+++) treatment.

Ectodermal factor p63 was expressed in all healthy controls. Moreover, high staining intensity (+++) was detected in 100% of the specimens. A significant reduction in the marker was found in the epithelium of patients with OLP (*p* < 0.05). Severe expression (+++) of the p63 protein was revealed in only 50% of the sections. Additionally, 30% of the specimens were p63 negative, and in another 10% this marker was expressed in less than 25% of the keratinocytes (+) (Figure 3). Brown nuclear staining for p63 was restricted to the basal/parabasal keratinocytes of the OLP epithelium (Figure 4).

According to statistical analysis, no significant difference in p63 expression was found between keratotic and non-keratotic forms of OLP (*p* = 0.39).

**Figure 5:**
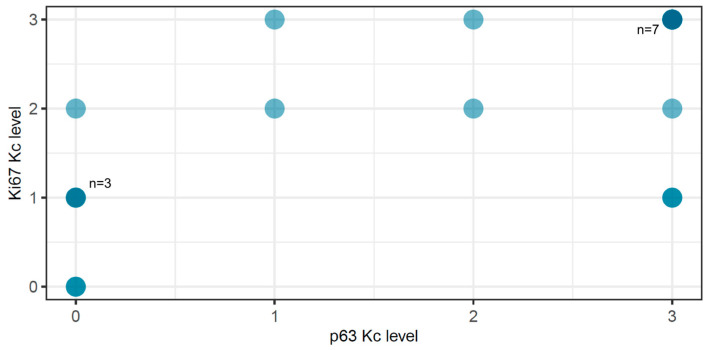
Association between p63- and Ki-67 levels (Spearman correlation test) in 20 patients with OLP. Light-blue dots represent a single case (patient) (*n* = 1), with corresponding p63 and Ki-67 levels shown; medium-blue dots represent two cases (patients) (*n* = 2); and dark-blue dots represent more than two cases (patients, *n* = 3 and *n* = 7), with p63 and Ki-67 levels shown.

Regarding Ki-67 expression in the same 20 OLP patients, a marked although not statistically significant (*p* = 0.28) reduction in Ki-67 staining intensity was found in OLP lesions compared to healthy mucosa. One-month treatment with an 810 nm diode laser resulted in an increase in the percentage of Ki-67-stained cells, which, however, was not statistically significant (*p* = 0.3). Ki-67 expression data were used in the present study to determine the association with the epidermal factor p63. The applied Spearman test demonstrated a significant correlation between p63 and Ki-67 expression in OLP patients (*p* = 0.001; 95% confidence interval 0.3149 to 0.8630; Spearman r 0.67) (Figure 5).

**Figure 6:**
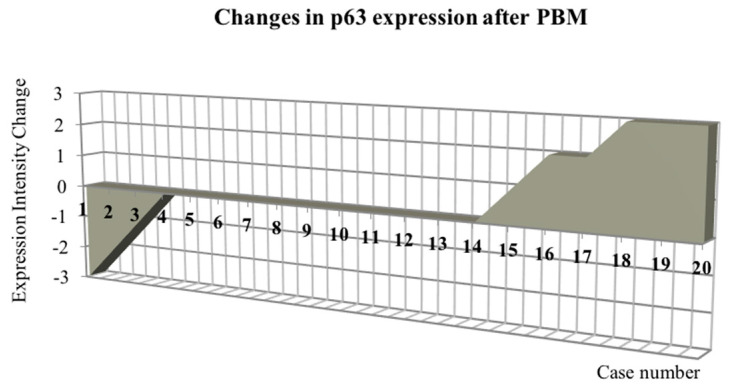
Change in p63 expression for all 20 OLP patients after PBM therapy.

Low-intensity laser irradiation (PBM) corrected the pre-treatment p63-deficiency in OLP tissues. The percentage of p63 positivity increased from 70% at baseline to 85% after PBM. An enhancement in the intensity of the immune reaction was also demonstrated. Figure 6 illustrates the change in p63 protein levels from those at baseline (“0” horizontal axis) for each of the patients included in the study. Two or three degrees of increase in p63 expression was observed in six cases, of which four had the non-keratotic and two had the keratotic form of OLP. A decrease in p63 levels following laser irradiation was established in three cases. Although markedly increased, p63 expression in OLP after PBM was not significantly different than that before therapy (*p* = 0.42). However, no significant difference was found between p63 immunoreactivity in OLP lesions after therapy and that in the healthy control group, either (*p* = 0.14). All cases of an increase in p63 expression after PBM therapy were accompanied by increased Ki-67 staining. In two of three cases that showed a decrease in p63-positive cells after therapy, concomitant decreased expression of the proliferative marker Ki-67 was also observed.

## 4. Discussion

In the conducted study, we found significantly decreased expression of the ectodermal factor p63 in OLP lesions compared with normal oral mucosa. Furthermore, data in the literature are relatively consistent regarding lower levels of the protein in these patients [21,22,23]. A few sources indicated increased expression [43]. Therefore, p63 deficiency can be hypothesized to be an important molecular mechanism in the pathogenesis of the disease.

Ebrahimi M et al. went even further, as they suspected that the p63 insufficiency could be an initiating factor for the disease progression. Here is their explanation: Epithelial cells in OLP lesions could not complete their differentiation due to a blockage of this essential -for the process marker and thus are considered “foreign” to the body and evoke an immune response [18]. This hypothesis could not answer why OLP also occurs in patients with strong expression of p63 in their oral epithelium. However, even if not the cause of the disease, lower p63 levels are part of the molecular perturbations seen in OLP. Given the leading functions of the protein, below we discuss the possible effects of the altered p63 expression on the integrity and the renewal of *tunica epithelialis*.

Continuous regeneration of the oral epithelium occurs thanks to a fine balance between cell proliferation provided by mitotically active keratinocytes in the basal layer and subsequent gradual differentiation of daughter cells as they migrate to the superficial stratum corneum. Poorly differentiated or undifferentiated cells were not detected in any of the OLP tissue specimens. Hence, the decreased expression of p63 does not appear to significantly affect the differentiation process in this disease. On the other hand, the presence of parakeratosis, representing a process of incomplete keratinization, raises the question of a possible defect in isoforms of p63 that are responsible for the late stages of maturation in the formation of the *stratum corneum*. In this regard, in their experiments, Truong A. et al. demonstrated that TAp63 isoforms contributed to later aspects of differentiation and that TAp63 knockdown resulted in incomplete development of the granular and corneum layers [16].

In vitro studies have shown that a lack of p63 resulted in severe epithelial hypoplasia [16]. To determine whether lower levels of p63 could affect the proliferative process in OLP, we looked for an association with Ki-67 and found that the two markers correlated significantly (*p* = 0.001) (Figure 4). Ki-67 is crucial in the proliferation process. Its expression begins in the G1 phase, increases gradually in the subsequent –S and G2 phases, and reaches its maximum during cell division (M phase) [44]. In eight out of ten cases with decreased levels of p63, a lower intensity of Ki-67 was also detected. Therefore, we can conclude that one of the effects of the altered expression of p63 in the pathogenesis of oral lichen planus is the suppression of cell proliferation. This could be the reason for the thinning of the epithelium in the atrophic–erosive form of the disease.

P63 is an apoptotic marker and apoptosis is the main pathological process in OLP. TAp63 isoforms induce apoptosis, while ΔNp63 inhibits programmed cell death [11]. It is thought that p63 is required for p53-mediated epithelial cell death [43]. According to Ebrahimi M et al., these two members of the p53 family act in a coordinated manner but are inversely correlated—low levels of p63 and high expression of p53 are needed to induce apoptosis [22]. The same association between these markers has been shown in UVA-induced apoptosis, which results from radiation-induced stabilization of p53 and a decrease in p63 [45,46]. Considering the above and based on the results found in this study, we may draw a conclusion that another effect of p63 deficiency in OLP is the activation of programmed cell death.

The absence of p63 leads to defects in adhesion, which results in cell death (anoikis) [47]. B-catenin, E-cadherin, and the epidermal growth factor receptor (EGFR) are p63-dependent proteins that play a crucial role in cell adhesion [21]. Consistent with the low levels of p63, decreased expression of all three markers was found in OLP lesions compared with healthy controls [21]. A lack of E-cadherin has been suggested to contribute to the basal cell degeneration seen in this disease [21]. However, in the present study, signs of anoikis were not detected in the samples. It is noteworthy that the only case of the bullous form in our OLP contingent was p63 negative.

From the above, it can be assumed that the expression of p63 is related to the clinical manifestation of the disease. Low levels of this marker should be expected in the atrophic, erosive, and bullous forms of OLP, given that p63 deficiency affects processes such as proliferation, apoptosis, and cell adhesion. However, we did not find a significant difference in protein levels between keratotic and non-keratotic forms of OLP—a lack of p63 was detected in three cases with the atrophic–erosive form, in one case with the bullous form, and in three cases with the keratotic form.

P63 is strongly associated with cancer development [10]. The protein has been shown to be overexpressed in both OSCC [9,23,48] and oral dysplastic lesions [9,49]. Pansini P et al. (2021) stated that p63 plays a role in oral tumorigenesis and represents promising biomarkers able to recognize mesenchymal phenotype induction in the transition from non-malignant cells to tumor cells [9]. This determines the value of the protein in the identification of oral lesions with a high risk for transformation to OSCC. Additionally, Sundberg et al. reported that overexpression of p63 and especially the combination of overexpression of both p63 and p53 were significantly associated with a higher recurrence risk of oral leucoplakia [10]. Therefore, assessment of p63 levels may be applied as a routine diagnostic test in all premalignant conditions, as it may reduce cancer-related mortality and morbidity. OLP is also included in the group of OPMDs. In the present study, we found lower expression of p63 in OLP lesions compared with normal oral mucosa, which is consistent with data in the literature [21,22,23]. Furthermore, p63 staining was observed only in the basal and parabasal keratinocytes of OLP lesions, as was also the case in the healthy controls’ mucosa. In the dysplastic epithelium, p63 expression has been shown to extend to the suprabasal layers [49]. Based on these findings, we can conclude that there are no signs of malignant transformation in the lesions of the studied OLP contingent.

As a limitation of the conducted immunohistochemical analysis, it should be emphasized that the anti-p63 antibody used detected both TAp63 and ΔNp63 isoforms, and thus, both were indistinguishably visualized as brown nuclear staining. Delineation of these two isoforms of p63 is important, as they have different, often opposing effects. In this regard, the lack of these data limits interpretation of the results.

When the p63 immunohistochemical profile of the OLP patients included in the study was established, they all underwent PBM laser therapy for one month. The treatment protocol was determined based on the results of our previous in vitro experiment regarding 810 nm laser dosimetry, where we found that the applied laser parameters (0.5 W; 30 s; 1.2 J/cm^2^) led to a significant reduction in the levels of pro-inflammatory cytokines (IL -1β, L -6, and IL-10) and to proliferation of fibroblast cell lines (McCoy–Plovdiv) [50]. Patient complaints ranged from mild discomfort to excruciating pain, disturbing food and beverage intake. The laser irradiation significantly alleviated the symptoms of the disease.

At baseline, different types of oral lesions were recorded—keratotic lines, papules and plaques, atrophic fields, erosions, and blisters. According to the TSSS, a significant improvement in clinical signs was achieved after PBM.

The treatment efficacy index as a function of symptomatic relief and oral lesion improvement was calculated for each of the OLP patients. The obtained results showed the effectiveness of PBM therapy in 90% of the cases. Atrophic and erosive lesions of OLP demonstrated better treatment outcomes compared with keratotic ones.

Deep insight into the tissue effects of PBM could provide an explanation for the above-mentioned finding. The recovery of the reticular-, papular-, and plaque-like forms of OLP requires keratolytic therapy, while the atrophic–erosive form can be improved by accelerating the regenerative processes. According to the scientific literature, PBM does not have a keratolytic action; wound healing, one the other hand, is a well-known effect of this kind of therapy. The rationale for the exertion of this effect includes several mechanisms of action, such as increased growth hormone secretion; activation of the TGF-β cytokine family (transforming growth factor-β); promotion of the proliferation of fibroblasts, keratinocytes, osteoblasts, and chondrocytes; matrix synthesis; angiogenesis; and vascular remodeling [29,33,51]. Recently, it has been reported that PBM enhanced reepithelialization of skin defects on rats by upregulating the protein expression of P63 [40]. This finding, together with data in the literature regarding the diminished p63 levels in patients suffering from oral lichen planus, made it interesting to investigate the potential of PBM to treat OLP lesions by influencing this molecular disturbance.

PBM therapy partially corrected the established p63 deficiency in OLP lesions. P63-positive cases as well as staining intensity for the marker increased at the end of the one-month course of treatment, although no statistically significant difference was found between pre- and post-treatment levels of protein expression. On the other hand, however, p63 immunoreactivity in OLP lesions after therapy did not differ significantly from that in the healthy control group, either (*p* = 0.14). This means that PBM therapy can increase the expression of epidermal factor p63 to a level close to that in normal oral mucosa. Further studies with an increased sample size are needed to confirm the validity of the results obtained. P63 change was accompanied by an increase in Ki-67 expression, and again, an association between these two markers was found—all cases of p63 elevation demonstrated concomitant increase of the proliferative Ki-67. This may explain the established increased improvement in the atrophic–erosive form of OLP compared to the keratotic one.

The acceleration of wound repair achieved with PBM has been well documented and involves the proliferation of fibroblasts and keratinocytes. However, the mechanisms by which low-power laser irradiation induces proliferation remain unclear. In the present study, we linked the observed clinical improvement of OLP lesions with an increase in the expression of epidermal factor p63 and the associated marker Ki-67. The mechanisms by which PBM therapy upregulates p63 expression can be speculated on. The main target of near-infrared (810 nm) phototherapy is the mitochondria. It is thought that photons are absorbed by mitochondrial chromophores in cells and increase reactive oxygen species, adenosine triphosphate, nitric oxide release, and blood flow, and activate diverse signaling pathways [33]. The p63 gene, on the other hand, maps to chromosome 3q27 [14]. Several signaling pathways and transcription factors have been identified that regulate p63 expression. Some of them (NF-kB, Notch, and Hedgehog signaling) repress while others (Wnt and EGFR) activate p63 [14]. Moreover, most of these regulative mechanisms appear to be isoform- and cell-type-specific. Further studies are needed to explore the complex network of interactions leading to p63 expression.

There is a group of diseases, such as CUS and GVHD, that present clinically with erosions or ulcers and mimic the histopathological features of oral lichen planus. The levels of epidermal factor p63 have been reported to be downregulated in these two conditions [22,52]. Therefore, it can be speculated that PBM will also be effective in the management of this patient contingent.

## 5. Conclusions

The observed significant reduction in epidermal factor p63 in the epithelium of patients with oral lichen planus is likely to be an important molecular mechanism in the pathogenesis of the disease. One of the possible effects of this protein insufficiency is the suppression of cell proliferation, as an associated decrease in Ki-67 was also found in these cases. These molecular disturbances predispose to thinning of the epithelium, erosions, and ulcers. PBM therapy with an 810 nm diode laser significantly improved the symptoms and clinical signs of OLP and upregulated the expression of p63, which was also accompanied by increased staining for the proliferation marker Ki-67. This could explain the increased improvement in the atrophic–erosive form of OLP compared to the keratotic one.

## Figures and Tables

**Table 1:** Clinical characteristics of patients with oral lichen planus (OLP) (n = 20).

Gender		Age		Clinical form		Localization	
Variable	n (%)	Variable	n (%)	Variable	n (%)	Variable	n (%)
male	3 (15%)	<30	1 (5%)	reticular	6 (30%)	buccal mucosa	14 (70%)
female	17 (85%)	31–40	2 (10%)	papular	1 (5%)	tongue	7 (35%)
		41–50	7 (35%)	plaque-like	2 (10%)	labial mucosa	4 (20%)
		51–60	3 (15%)	atrophic	5 (25%)	gingiva	10 (50%)
		>61	7 (35%)	erosive	5 (25%)	palate	2 (10%)
				bullous	1 (5%)		

## Data Availability

All the data are provided in the text.
